# New biplane x-ray magnetic resonance image fusion prototype for 3D enhanced cardiac catheterization in congenital heart diseases

**DOI:** 10.1186/1532-429X-16-S1-O103

**Published:** 2014-01-16

**Authors:** Tanja Kurzendorfer, Erin Girard, Kevin Gralewski, Andreas Kleinoeder, Atilla P Kiraly, Norbert Strobel, Yoav Dori

**Affiliations:** 1 Imaging and Computer Vision, Siemens Corporate Technology, Princeton, New Jersey, USA; 2 Cardiology, Childrens Hospital of Philadelphia, Philadelphia, Pennsylvania, USA; 3 Imaging & Therapy Division, Siemens AG, Healthcare Sector, Forccheim, Germany

## Background

X-ray magnetic resonance fusion (XMRF) is used to enhance fluoroscopically guided catheterization procedures. We present a new method for biplane XMRF involving an augmented fluoroscopy prototype (Siemens, Forchheim, Germany). With this software it is possible to register 3D MRI data to biplane X-ray projections based on internal markers, without the need for a C-arm CT to perform 3D/3D registration. The software supports overlaying volume rendered data as well as multiple surface models. Visualization techniques, such as contour or solid rendering and surface carving are supported to allow for clear presentation of complex 3D structures.

## Methods

We reviewed data obtained with different visualization methods on 20 patients that underwent clinical XMRF procedures. Surface models were generated by threshold based segmentation from high resolution MRA (syngo Twist or Navigator gated 3D flash IR sequence) of structures of interest using Mimics (Leuven, Belgium). Initial registration was achieved through planar alignment of the volume in anterior-posterior (AP) and lateral projections matching anatomical landmarks, such as the heart and vessel borders. In addition, the registration accuracy of the prototype was assessed using a phantom by measuring the maximum distance between a single point and the 3D model boundaries in comparison to the boundaries seen on fluoroscopy.

## Results

**Figure 1:**
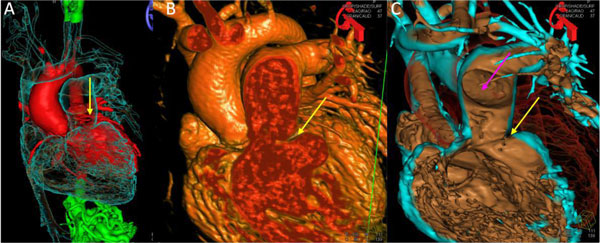
**A) Three surface models loaded; the right side of the heart (blue) is contour rendered, the left side of the heart (red) is solid rendered, allowing us to visualize the relationship between the stenotic pulmonary artery and the left coronary arteries (arrow)**. B) Volume rendered image with clip plane, showing the area of the stenosis (arrow). The relationship between the stenosis and the pulmonary artery ostia cannot be visualized. C) Solid rendered surface cut open using a "carving" technique to visualize the relationship between the stenosis (yellow arrow) and the right pulmonary artery ostium (purple arrow).

**Figure 2:**
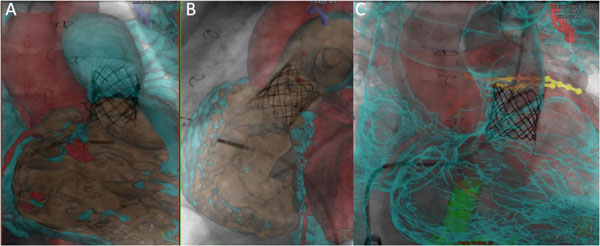
**A) Anterior-posterior, and B) lateral biplane X-ray magnetic resonance fusion with two surfaces loaded**. The right side of the heart is carved open to visualize the valve placement in the pulmonary artery. C) Contour rendered overlay of the right side of the heart in the cranial projection showing the spatial relation between the valve and the left coronary arteries which were marked with 3D lines to enhance visualization.

The maximum segmentation time was 10 min and the initial registration required less than 30 sec. Registration was performed without the need for contrast injection or additional radiation exposure. The registration error based on phantom measurements was 2.2 ± 1.1 mm in the AP projection and 1.36 ± 0.7 mm in the lateral projection. For surface rendered data, solid rendering with carving provided the optimal display of complex 3D data. In contrast to volume rendering, solid surface rendering provides delineation of the 3D relation between objects and clear visualization of internal structures such as ostia of the vessels and muscle bundles using the carving feature, see Figure 1. Contour rendering offered reasonable visualization for smooth, uncomplicated structures like the atria, but it was not ideal for complex overlapping structures due to missing 3D depth information. When multiple surfaces are loaded, contour rendering was, however, found to be useful in combination with solid rendered structures to provide a see-through 3D relation between multiple structures, see Figure 2.

## Conclusions

The prototype demonstrated a high level of accuracy for fluoroscopic overlays. Biplane internal marker based registration can be performed quickly, without the need for additional radiation or contrast. We found solid rendering of surface models in combination with carving techniques to be most useful for visualization. This new biplane XMRF technique has the potential to provide enhanced guidance under fluoroscopy by integrating 3D information and to reduce radiation during complicated catheterization procedures.

## Funding

This project is funded by a research grant from Siemens, AG, Healthcare, Forchheim, Germany. The concepts and information presented in this paper are based on research and are not commercially available.

